# Prolonged use of neuromuscular blocking agents is associated with increased long-term mortality in mechanically ventilated medical ICU patients: a retrospective cohort study

**DOI:** 10.1186/s40560-023-00696-x

**Published:** 2023-11-17

**Authors:** Chun Lin, Wen-Cheng Chao, Kai-Chih Pai, Tsung-Ying Yang, Chieh-Liang Wu, Ming-Cheng Chan

**Affiliations:** 1 Division of Chest Medicine, Department of Internal Medicine, Taichung Veterans General Hospital, Taichung, Taiwan; 2 Department of Critical Care Medicine, Taichung Veterans General Hospital, Taichung, Taiwan; 3 Department of Post-Baccalaureate Medicine, College of Medicine, National Chung Hsing University, Taichung, Taiwan; 4 Department of Automatic Control Engineering, Feng Chia University, Taichung, Taiwan; 5 Big Data Center, Chung Hsing University, Taichung, Taiwan; 6 College of Engineering, Tunghai University, Taichung, Taiwan; 7 Department of Life Sciences, National Chung Hsing University, Taichung, Taiwan; 8 Division of Critical Care and Respiratory Therapy, Department of Internal Medicine, Taichung Veterans General Hospital, Taichung, Taiwan

**Keywords:** Neuromuscular blockade, Mechanical ventilation, Long-term mortality

## Abstract

**Background:**

Neuromuscular blockade agents (NMBAs) can be used to facilitate mechanical ventilation in critically ill patients. Accumulating evidence has shown that NMBAs may be associated with intensive care unit (ICU)-acquired weakness and poor outcomes. However, the long-term impact of NMBAs on mortality is still unclear.

**Methods:**

We conducted a retrospective analysis using the 2015–2019 critical care databases at Taichung Veterans General Hospital, a referral center in central Taiwan, as well as the Taiwan nationwide death registry profile.

**Results:**

A total of 5709 ventilated patients were eligible for further analysis, with 63.8% of them were male. The mean age of enrolled subjects was 67.8 ± 15.8 years, and the one-year mortality was 48.3% (2755/5709). Compared with the survivors, the non-survivors had a higher age (70.4 ± 14.9 vs 65.4 ± 16.3, *p* < 0.001), Acute Physiology and Chronic Health Evaluation II score (28.0 ± 6.2 vs 24.7 ± 6.5, *p* < 0.001), a longer duration of ventilator use (12.6 ± 10.6 days vs 7.8 ± 8.5 days, *p* < 0.001), and were more likely to receive NMBAs for longer than 48 h (11.1% vs 7.8%, *p* < 0.001). After adjusting for age, sex, and relevant covariates, the use of NMBAs for longer than 48 h was found to be independently associated with an increased risk of mortality (adjusted HR: 1.261; 95% CI: 1.07–1.486). The analysis of effect modification revealed that this association was tended to be strong in patients with a Charlson Comorbidity Index of 3 or higher.

**Conclusions:**

Our study demonstrated that prolonged use of NMBAs was associated with an increased risk of long-term mortality in critically ill patients requiring mechanical ventilation. Further studies are needed to validate our findings.

## Background

Mechanical ventilation (MV) is a relevant organ support system and is increasingly used in intensive care units (ICUs) worldwide [1–3]. Asynchrony is common in critically ill patients requiring MV and consists of reverse triggering [4] with breath stacking. This can potentially cause diaphragmatic injury, especially in patients with acute respiratory distress syndrome (ARDS) [5]. The use of neuromuscular blockade agents (NMBAs) has been found to reduce asynchrony and short-term mortality in patients with moderate-to-severe ARDS [6, 7].

However, increasing evidence has identified that the use of NMBAs may lead to deleterious impacts, including disuse atrophy of the diaphragm and ICU-acquired weakness [8–10]. The conflicting evidence regarding the role of NMBAs highlights the crucial need to investigate the long-term impact of NMBAs on mortality in critically ill ventilated patients.

In the present study, we linked data from the Taiwanese National Health Insurance Research Database (NHIRD) and the critical care database at Taichung Veterans General Hospital (TCVGH) to examine the relationship between the use of NMBA and long-term mortality in critically ill ventilated patients. We hypothesized that the infusion of NMBA would associate with a higher one-year mortality rates in this group of patients.

Part of the results from the current study have been accepted as an abstract for a poster presentation at the 2023 ATS International Conference (May 19–24, 2023, Washington, DC).

## Methods

### Subjects and data collection

We conducted a retrospective cohort study at TCVGH, a tertiary care teaching hospital located in central Taiwan with approximately 1500 beds. We collected data on consecutive adult patients who were admitted to the medical ICUs and received MV between 2015 and 2020. Patients who used NMBAs continuously for more than 8 h within the first 72 h of ICU admission were included in the analysis. We considered the recent clinical practice guidelines and selected a time frame of 48 h for patient grouping [11–13].

Demographic characteristics of the patients included in the study, such as age, sex, Charlson Comorbidity Index (CCI), etiology for ICU admission, Acute Physiology and Chronic Health Evaluation (APACHE) II score, Sequential Organ Failure Assessment (SOFA) scores, laboratory data, and ventilatory parameters of mechanical ventilation, were obtained from the TCVGH clinical data warehouse. We obtained the discharged patients’ date of death from the nationwide death registration profile of NHIRD in Taiwan.

We excluded patients who died within 72 h after ICU admission to ensure that we had complete data for the subjects enrolled in this study. In patients with more than two ICU admissions, we defined the first ICU admission as the index ICU admission.

### Outcome

The primary outcome of interest was the time to one-year all-cause mortality following admission to the ICU. Given that the National Health Insurance (NHI), a compulsory program, has been implemented in Taiwan since 1995, with nearly 99.9% coverage of the population in 2019 [14], the date of death among enrolled patients in this study should be accurate.

### Statistical analyses

Data for categorical variables are presented as numbers (percentages), while data for continuous variables are shown as means ± standard deviation. Kaplan–Meier analysis was conducted to analyze the association between long-term mortality and the use of NMBA. We conducted a Cox proportional hazards model to estimate hazard ratios (HRs) and 95% confidence intervals (CIs) for one-year all-cause mortality after adjustment for potential confounders such as age, gender, CCI, etc. Statistical analyses were two-sided, and the level of significance was set at 0.05.

We also utilized the Wald test to examine the modification effect by covariates, to assess whether the magnitude of association between the use of NMBAs and long-term mortality differed based on clinical variables such as age, sex, body mass index (BMI), CCI, APACHE II, FIO2 (fraction of inspired oxygen), and PEEP (positive end-expiratory pressure).

### Ethics approval

The study was conducted in accordance with the Declaration of Helsinki. This study was approved by the Institutional Review Board of TCVGH with a waiver of informed consent since it was a retrospective analysis of anonymous data (IRB number: SE20249B).

## Results

### Patient enrollment and characteristics

**Fig. 1:**
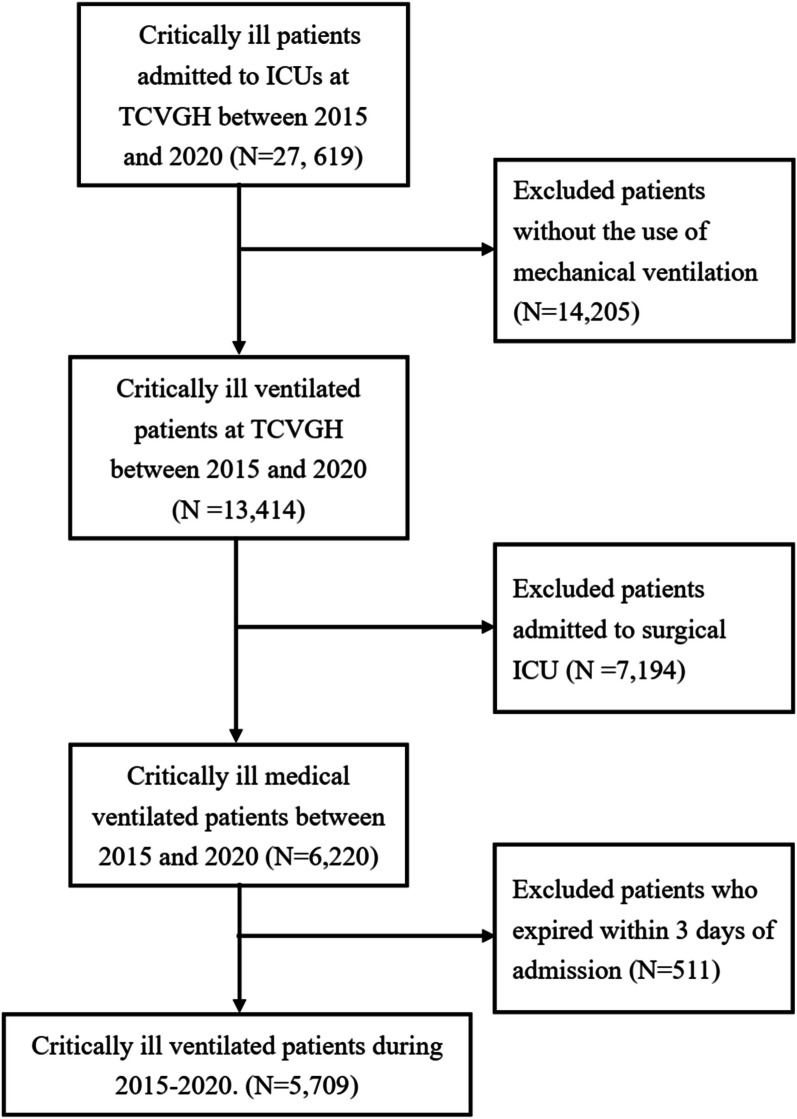
Flowchart of the subject enrollment

Figure 1 shows the process of patient enrollment. A total of 27,619 patients who were admitted to the ICUs from January 2015 to December 2019 were included. Given that we focused on critically ill patients receiving mechanical ventilation for more than 2 days in medical ICUs, we excluded the patients who were admitted to the surgical ICU (*n* = 1634), underwent cardiovascular surgery (*n* = 5560), and those who died within 3 days after admission (*n* = 511). A total of 5709 patients were eligible for further analysis, with their baseline characteristics summarized in Table 1.

**Table 1:** Characteristics of the enrolled critically ill patients divided by overall mortality

Variables	All (*n* = 5709)	Survivors (*n* = 2954)	Non-survivors (*n* = 2755)	*p* value^a^
Basic characteristics				
Age, years	67.8 ± 15.8	65.4 ± 16.3	70.4 ± 14.9	< 0.001
Sex (male)	3645 (63.9%)	1815 ( 61.4%)	1830 (66.4%)	< 0.001
Body mass index	24.1 ± 5.0	24.7 ± 5.0	23.5 ± 5.0	< 0.001
Charlson Comorbidity Index	3.8 ± 2.6	3.0 ± 2.3	4.50 ± 2.7	< 0.001
Follow-up duration, years	1.9 ± 2.0	3.4 ± 1.7	0.2 ± 0.3	< 0.001
Etiology for ICU admission				
Acute respiratory failure	2469 (43.2%)	1183 (40.1%)	1286 (46.7%)	< 0.001
Pneumonia	658 (11.5%)	287 (9.7%)	371 (13.5%)	< 0.001
Sepsis (non-pneumonia)	562 (9.8%)	252 (8.5%)	310 (11.3%)	0.001
Acute cardiac conditions	178 (3.1%)	109 (3.7%)	69 (2.5%)	0.012
Acute neurological conditions	558 (9.8%)	328 (11.1%)	230 (8.4%)	0.001
Acute renal conditions	224 (3.9%)	115 (3.9%)	109 (4.0%)	0.956
Acute GI condition	287 (5.0%)	133 (4.8%)	154 (5.4%)	0.069
Post-PCI	45 (0.8%)	30 (1.0%)	15 (0.5%)	0.063
Cardiac arrest (IHCA + OHCA)	68 (1.2%)	29 (1.0%)	39 (1.4%)	0.165
Others	732 (11.6%)	380 (12.9%)	352 (12.8%)	0.953
Severity and managements				
APACHE II score	26.4 ± 6.6	24.7 ± 6.5	28.0 ± 6.2	< 0.001
SOFA score, day-1	8.5 ± 3.6	7.8 ± 3.4	9.3 ± 3.6	< 0.001
SOFA score, day-3	7.5 ± 3.9	6.3 ± 3.3	8.6 ± 4.0	< 0.001
SOFA score, day-7	6.7 ± 3.8	5.2 ± 2.8	7.9 ± 4.1	< 0.001
Laboratory data				
White blood cell count (count/μl)	11,455 ± 9849	10,720 ± 5794	12,240 ± 12,790	< 0.001
Hemoglobin (g/dL)	10.0 ± 2.0	10.5 ± 2.0	9.6 ± 1.8	< 0.001
Platelet (10^3^/μL)	174.9 ± 109.2	198.0 ± 105.2	150.3 ± 108.0	< 0.001
Creatinine (mg/dL)	2.1 ± 2.2	1.9 ± 2.4	2.2 ± 2.0	< 0.001
Lactate (mg/dL)	17.0 ± 18.3	13.7 ± 9.2	20.3 ± 23.8	< 0.001
Ventilatory data				
Ventilator days	10.1 ± 9.8	7.8 ± 8.5	12.6 ± 10.6	< 0.001
FIO_2_ (%)	50.7 ± 21.2	48.9 ± 20.2	52.4 ± 22.0	< 0.001
PaO_2_: FIO_2_ ratio	270.8 ± 183.9	277.2 ± 191.2	264.0 ± 175.7	0.018
PEEP (cmH_2_O)	7.9 ± 4.6	7.6 ± 4.2	8.2 ± 4.9	< 0.001
P_peak_, (cmH_2_O)	26.4 ± 6.6	25.8 ± 6.5	27.1 ± 6.6	< 0.001
P_mean_, (cmH_2_O)	14.3 ± 4.9	13.8 ± 4.5	14.8 ± 5.2	< 0.001
V_T_ (L)	0.57 ± 0.16	0.58 ± 0.16	0.56 ± 0.16	< 0.001
Minute ventilation (L)	10.7 ± 3.2	10.7 ± 3.1	10.8 ± 3.2	0.735
PaO_2_ (mmHg)	163.1 ± 101.5	163.0 ± 104.0	163.1 ± 100.2	0.988
PaCO_2_ (mmHg)	39.5 ± 13.4	39.8 ± 13.1	39.3 ± 13.7	0.197
HCO_3_^−^ (mmol/l)	22.3 ± 6.3	22.4 ± 5.9	22.3 ± 6.6	0.356
Use of NMBAs				
None	4409 (77.2%)	2332 (78.9%)	2077 (75.4%)	< 0.001
Use, equal or less than 48 h	765 (13.4%)	394 (13.3%)	371 (13.5%)	
Use, longer than 48 h	535 (9.37%)	228 (7.8%)	307 (11.1%)	
Outcomes				
ICU-stay, days	12.7 ± 10.0	10.7 ± 8.6	14.8 ± 10.9	< 0.001
Hospital-stay, days	28.3 ± 24.2	26.5 ± 24.2	30.3 ± 24.0	0.002

*GI* gastrointestinal, *PCI* percutaneous coronary intervention, *IHCA* in-hospital cardiac arrest, *OHCA* out-of-hospital cardiac arrest, *APACHE II* Acute Physiology and Chronic Health Evaluation, *SOFA* score: Sequential Organ Failure Assessment (SOFA) Score, *FIO*_*2*_, fraction of inspired oxygen, *PaO*_*2*_ FIO_2_ ratio: ratio of partial pressure of arterial oxygen to inspired oxygen fraction, *PEEP* positive end-expiratory pressure, *P*_*peak*_ peak airway pressure, *PaO*_*2*_ partial pressure of arterial oxygen, *PaCO*_*2*_ partial pressure of arterial carbon dioxide, *HCO*_*3*_ serum bicarbonate, *V*_*T*_ tidal volume, *ICU* intensive care unit, *NMBAs* neuromuscular blocking agents

^a^*p* value represents comparisons between the survivors and non-survivors critically ill patients

The mean age of the enrolled patients was 67.8 ± 15.8 years, and 63.8% of them were men. The most common etiology for ICU admission was acute respiratory failure (*n* = 2469, 43.2%), followed by pneumonia (*n* = 658, 11.5%) and sepsis other than pneumonia (*n* = 562, 9.8%). Regarding the severity of the patients, the mean APACHE II score was 26.4 ± 6.6, and the mean SOFA score was 8.5 ± 3.6 on the first day of ICU admission. The overall in-hospital mortality rate was 27.6%, with 90-day and 1-year mortality rates of 34.2% and 48.3%, respectively. The 1-year mortality rate of patients who received NMBAs infusion for more than 48 h was 57.4%.

We further divided critically ill ventilated patients based on their 1-year mortality. As compared to the survivors, non-survivors were older and more frequently male. They were also more commonly admitted due to acute respiratory failure or pneumonia. The non-survivor also had a higher CCI, APACHE II score, and day-1/3/7 SOFA scores, as well as a lower BMI than those in the survivor group.

## The use of NMBAs and ventilatory parameters among enrolled patients

The mean ventilator days of the included patients was 10.1 ± 9.8 days, while the mean FIO2 at ICU admission was 50.7 ± 21.0%, and the mean PEEP was 7.9 ± 4.6 (cmH_2_O). Non-survivors had a higher FIO_2_ (48.9 ± 20.2% vs 52.4 ± 22.0%, *p* < 0.001) and a higher PEEP (7.6 ± 4.2 cmH_2_O vs 8.2 ± 4.9 cmH2O, *p* < 0.001) at ICU admission. On the other hand, the survivors were more likely to have shorter ventilator days (7.8 ± 8.5 vs 12.6 ± 10.6 days, *p* < 0.001).

In terms of the use of NMBAs, 13.4% (*n* = 765) of the enrolled patients underwent NMBA treatment for equal or less than 48 h, while 9.37% (*n* = 535) of them received infusions for more than 48 h. In contrast to non-survivors, a higher number of survivors did not receive NMBAs infusion during ICU admission.

## Association between the use of NMBAs and long-term mortality in critically ill ventilated patients

**Fig. 2:**
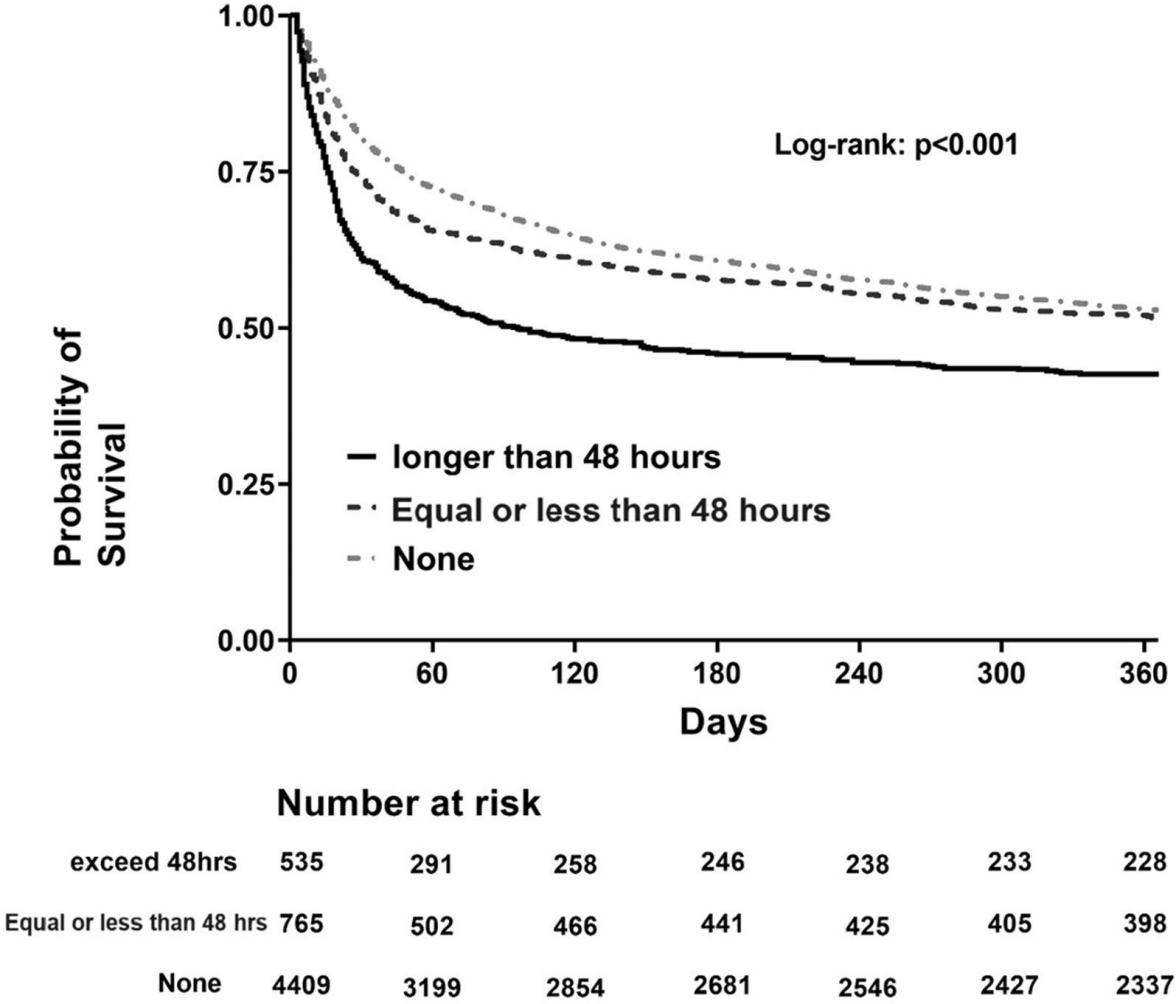
Kaplan–Meier survival curves among in critically ill ventilated patients stratified by the use of NMBAs

We conducted Kaplan–Meier analyses to demonstrate the association between mortality and the use of NMBAs, categorized as none, use for equal or less than 48 h, and use for longer than 48 h (Fig. 2). The multivariable Cox proportional hazard regression model found that age, gender (male), high CCI, high APACHE II, lower platelet count, high blood lactate level, and high demand for FIO_2_ (≥ 50%) were independently associated with mortality. Conversely, a high BMI was found to be a protective factor for long-term mortality in these patients (Table 2). We found that the use of NMBA for more than 48 h was independently associated with a higher 1-year mortality (HR: 1.261; 95% CI: 1.07–1.486) after adjusting for relevant covariates, including age, sex, BMI, comorbidities, APACHE II score, blood platelet and lactate level, etiologies of ICU admission, high demand for FIO2 (≥ 50%), PaO_2_ and high PEEP (≥ 8 cmH_2_O). The use of NMBAs for equal or less than 48 h appeared to be associated with long-term mortality, but this correlation became insignificant after adjusting for covariates.

**Table 2:** Cox regression for long-term mortality

Variables	Univariable		Multivariable	
	HR (95% C.I.)	*p* value^a^	HR (95% C.I.)	*p* value^b^
Age, per 1 year increment	1.013 (1.01–1.015 0)	< 0.001	1.006 (1.002–1.009 )	0.001
Male gender	1.160 (1.072–1.255)	< 0.001	1.179(1.066–1.303)	0.001
BMI, per 1 decrement	0.965 (0.957–0.973)	< 0.001	0.966 (0.956–0.976)	< 0.001
CCI, per 1 increment	1.165 (1.150–1.181)	< 0.001	1.150 (1.131–1.170)	< 0.001
APACHE II, per 1 increment	1.064 (1.057–1.071)	< 0.001	1.023 (1.014–1.032)	< 0.001
Platelet, per 1 × 10^3^ decrement	0.996 (0.995–0.996)	< 0.001	0.997 (0.997–0.998)	< 0.001
Lactate, per 1 increment	1.02 (1.019–1.0220)	< 0.001	1.028 (1.025–1.030)	< 0.001
Etiology for ICU admission				
Acute respiratory failure	1.201 (1.115–1.295)	< 0.001	1.048 (0.932–1.177)	0.434
Pneumonia	1.298 (1.163–1.448)	< 0.001	1.087 (0.941–1.256)	0.256
Sepsis (non-pneumonia)	1.289 (1.145–1.45)	< 0.001	0.999 (0.854–1.168)	0.988
Acute cardiac conditions	0.727 (0.572–0.923)	0.009	0.681 (0.415–1.116)	0.128
Acute neurological conditions	0.809 (0.707–0.926)	0.002	1.055 (0.880–1.265)	0.560
Ventilatory parameters				
FIO_2_ ≥ 50%	1.384 (1.282–1.493)	< 0.001	1.167 (1.050–1.297)	0.004
PEEP ≥ 8 cmH_2_O	1.366 (1.267–1.472)	< 0.001	0.939 (0.839–1.051)	0.275
PaO2, per 10 decrement	0.999 (0.995–1.003)	0.677	0.998 (0.993–1.002)	0.320
Use of NMBAs				
None	[Reference]		[Reference]	
Use, equal or less than 48 h	1.092 (0.978–1.220)	0.118	0.962 (0.834–1.111)	0.602
Use, longer than 48 h	1.506 (1.336–1.697)	< 0.001	1.261 (1.070–1.486)	0.006

*BMI* body mass index, *CCI* Charlson Comorbidity Index, *PaO2* partial pressure of arterial oxygen, *NMBAs*, neuromuscular blocking agents

To further investigate the potentially distinct magnitudes of the mortality impact of NMBAs among different patient subgroups, we conducted an analysis of effect modification. We found that the association between the use of NMBAs and mortality appeared to be stronger in patients with a CCI equal to or higher than 3 (Table 3).

**Table 3:** Effect modification of variables on the association between use of NMBAs and risk of mortality

Variables	Crude HR (95%CI)	*p* value for interaction	Adjusted HR (95% CI)	*p* value for interaction
Age				
< 65 years	1.436 (1.200–1.717)	0.279	1.026 (0.772–1.220) (0.772–1.220)	0.110
≥ 65 years	1.804 (1.530–2.126)		1.307 (1.075–1.590) (1.075–1.590)	
Gender				
Female	1.650 (1.333–2.044)	0.420	1.429 (1.093–1.868)	0.321
Male	1.419 (1.227–1.640)		1.070 (0.896–1.277)	
BMI				
< 18	1.378 (0.830–2.286)	0.050	1.558 (0.884–2.745)	0.248
≥ 18	1.559 (1.376–1.766)		1.131 (0.970–1.318)	
CCI				
< 3	1.272 (1.021–1.586)	0.010	1.038 (0.733–1.282)	0.040
≥ 3	1.829 (1.585–2.110)		1.205 (1.012–1.435)	
APACHE II				
< 25	1.073 (0.815–1.412)	0.344	1.136 (0.845–1.528)	0.361
≥ 25	1.342 (1.172–1.537)		1.282 (1.095–1.500)	
FiO_2_ (%)				
< 50	1.003 (0.814–1.235)	0.010	1.080 (0.852–1.369)	0.160
≥ 50	1.289 (1.090–1.525)		1.184 (0.965–1.452)	
PEEP (cmH_2_O)				
< 8	1.215 (0.772–1.912)	0.927	2.119 (1.227–3.659)	0.230
≥ 8	1.119 (1.047–1.367)		1.100 (0.940–1.289)	

*BMI* body mass index, *CCI* Charlson Comorbidity Index, *NMBAs* neuromuscular blocking agents

## Discussion

In the current study, we found that the use of NMBAs for more than 48 h may be associated with a higher 1-year mortality rate in critically ill medical patients requiring mechanical ventilation. Moreover, we noted that the mortality impact of NMBAs tends to be stronger in critically ill patients with multiple comorbidities. These findings provide clinical evidence regarding the prolonged detrimental impact of using NMBAs in critically ill ventilated medical patients.

Notably, the clinical evidence of the role of NMBAs varies depending on the study period. Two landmark randomized controlled trials, ACURASY [6] and ROSE [15] trials, were conducted to clarify the mortality benefit of using NMBAs on patients with ARDS. The ACURASYS trial found that in patients with ARDS and a PaO_2_:FIO_2_ ratio less than 150, early administration of NMBAs for 48 h improved the 90-day mortality. The ROSE trial, in contrast, found no difference in hospital mortality among ARDS patients with or without NMBAs. The conflicting results from these two extensive randomized controlled trials may be attributed to differences in patient enrollment and treatment approaches, including variations in PEEP level and depth of sedation. Therefore, the current international guidelines [11–13] suggested use of NMBAs in selected conditions. For example, they may be used in moderate-to-severe ARDS patients who required continuous deep sedation or to manage overt shivering in patients needing therapeutic hypothermia. The real-world finding from our study further provides clinical evidence of prolonged impact of the use of NMBAs.

The long-term outcome among patients receiving mechanical ventilation in ICU had been a topic of interest for years. The patients who required mechanical ventilation had a higher ICU mortality up to 17–28% [16, 17]. What is more, the use of mechanical ventilation also impacts long-term function and mortality, and those patients who required prolonged ventilation had considerably high long-term mortality [18–20]. Similarly, in the current study, additional 20.7% of the included patient died within 1 year after discharge, and patients who received NMBAs infusion for more than 48 h had an even higher 1-year mortality rate. In the case of ARDS, studies have shown a 1-year mortality rate range from 11% to 58% [21–23]. One Canadian multihospital cohort reported a 3-year mortality rate of 49.3% [24]. Another multihospital study from Baltimore found that 34% of the ARDS survivors died within 5 years after discharge [25]. Worsened long-term quality of life and reduced physical function were found among ARDS survivors as well [26]. Several studies had been carried out to examine the impact of NMBAs on long-term outcome of ARDS patients. Three meta-analyses [27–29] including previously published RCTs (randomized controlled trials), included the 2 large RCTs (ACURASYS and ROSE) mentioned above, had not found that NMBA infusion could improve 90-day mortality. Li Bassi, G. et al. conducted propensity score analysis using the dataset from a multinational multicenter cohort to investigate the effect of NMBA infusion in COVID-19 (coronavirus disease 2019) ARDS patients [30]. They reported that no association between 90-day mortality and with NMBA use (median duration: 6 days) was found after adjusting for covariates.

The long-lasting impact of NMBAs use may be associated with a number of potential mechanisms, including ICU-acquired weakness and diaphragm disuse atrophy. Prior studies have found that the presence of ICU-acquired weakness in ICU patients is associated with both short-term [31] and long-term mortality up to 5 years [25, 32]. A number of studies have shown the association between the use of NMBAs and the risk of developing ICU-acquired weakness. Price DR et al., in their analysis of 2,254 critically ill patients from 18 studies, found that the use of NMBAs modestly associated with the development of neuromuscular dysfunction in critical illness (odds ratio 1.25; 95% CI 1.06–1.48) [32]. Another meta-analysis conducted by T Yang et al. showed that the use of NMBAs had a significant association with ICU-acquired weakness [33]. Diaphragm atrophy had been reported within 24 h of mechanical ventilation and may related to worse outcomes, including prolonged ICU admission and a lower probability of weaning from the ventilator [34, 35]. However, there was a scarcity of real-world clinical data regarding the impact of NMBA on diaphragm function. Animal models found varied effects on diaphragm function with two NMBAs: while rocuronium exacerbated diaphragm weakness, cisatracurium did not demonstrate the same effects [36]. To sum up, further studies are needed to determine whether the use of NMBAs could potentially contribute to the development of ICU-acquired weakness and diaphragm dysfunction, and subsequently affect long-term outcomes.

In our study, we found that the use of NMBA for longer than 48 h was associated with increased 1-year mortality in critically ill ventilated patients. Furthermore, the analysis of effect modification showed that the strength of association between the use of NMBAs and poor long-term outcomes appears to be higher among patients with more comorbidities (CCI higher or equal to 3). Previously, multiple comorbidities had been considered as a risk factor for ICU-acquired weakness and increased mortality in ventilated patients [19, 37]. Similar to our finding, Pfoh ER et al. conducted a multihospital prospective cohort study and found that comorbidities were associated with a greater long-term physical decline in 193 ARDS survivors [38]. Clinicians should exercise caution when using NMBAs for an extended period in patients with multiple comorbidities, considering the existing data.

The current study has several limitations. First, this study was conducted at a single center, so the findings may not be applicable to other healthcare systems. However, the data were obtained from daily critical care, and the drawback of generalization should be partly reduced. Second, we excluded the patients who admitted to surgical ICU. Inevitably this might further mitigate the generalizability. But by excluding the patients who required post-operative care, we could possibly enhance the homogeneity of studied population. Third, some unmeasured cofounders, such as use of corticosteroid, measure of ICU-acquired weakness and rehabilitation, may exist in this study. Fourth, the dose of the NMBA was not recorded in the databases used in the current study, it is plausible that the dose of NMBAs may affects the outcomes as well. Fifth, the assessment related to diaphragmatic functions was not performed in the facility. Thus, we could not quantify the impacts of NMBAs on diaphragm from the current data.

## Conclusion

In the present study, we found that the administration of NMBAs for more than 48 h was associated with an increase in 1-year mortality among mechanically ventilated medical patients. Our findings highlight the risks of prolonged use of NMBAs in critical care patients. Further studies are warranted to clarify the possible mechanism and verified our findings.

## Data Availability

The data underlying this article will be shared on request to the corresponding author.

## Abbreviations

APACHE: Acute Physiology and Chronic Health Evaluation
ARDS: Acute respiratory distress syndrome
BMI: Body mass index
CCI: Charlson Comorbidity Index
CI: Confidence interval
COVID-19: Coronavirus disease 2019
FIO_2_: Fraction of inspired oxygen
HR: Hazard ratio
ICU: Intensive care unit
NHI: National Health Insurance
NHIRD: National Health Insurance Research Database
NMBA: Neuromuscular blocking agent
PaO_2_: Partial pressure of arterial oxygen
PaO_2_2FIO_2_ ratio: Ratio of partial pressure of arterial oxygen to inspired oxygen fraction
RCT: Randomized controlled trial
SOFA: Sequential Organ Failure Assessment
TCVGH: Taichung Veterans General Hospital
